# Hydrogen Peroxide Enema-induced Proctitis in a Young Female: A Case Report

**DOI:** 10.7759/cureus.6468

**Published:** 2019-12-26

**Authors:** Bayarmaa Mandzhieva, Muzammil Khan, Mamoon Ur Rashid, Rima Shobar, Abu H Khan

**Affiliations:** 1 Internal Medicine, AdventHealth, Orlando, USA; 2 Internal Medicine, Khyber Teaching Hospital, Peshawar, PAK; 3 Gastroenterology, AdventHealth, Orlando, USA

**Keywords:** hydrogen peroxide, enema, chemical colitis, proctitis

## Abstract

Hydrogen peroxide is a disinfectant commonly used for cleansing superficial wounds due to its oxidizing capacity. In the past, it has also been used for the management of meconium ileus in children as the oxidizing action of hydrogen peroxide potentiates peristalsis that relieves ileus or fecal impaction. The potential dangers were unknown till Pumphrey, in 1951, described the harmful effects of its use as an enema. We present a case of a 32-year-old female who was admitted for complaints of perianal pain. She used an enema, consisting of water and hydrogen peroxide, for constipation. It improved her symptoms but subsequently, she developed a burning sensation in her rectum. The patient had a colonoscopy which revealed severe proctitis up to 15 cm from the anal verge manifested by superficial mucosal ulceration, marked erythema, and edema with friable mucosa and hemorrhage. The patient was subsequently given mesalamine, and the symptoms resolved.

## Introduction

For many years, hydrogen peroxide was known as a home panacea for the management of meconium ileum in the pediatric population and fecal impaction in the elderly because it initiated peristaltic reflex. Even the review of old articles until the 1950s will reveal multiple recommendations and physician practices in terms of different percent solutions and regimens [1]. Pumphrey, one of the first, in 1951, recognized its detrimental effects and described the caustic action and potential dangers of hydrogen peroxide enema [2]. During the 1980s, hydrogen peroxide was used for disinfection of endoscopes and several cases of hydrogen peroxide colitis were reported. Multiple reports of the sequelae related to rectal administration were disclosed after that [3].

This case is important because it shows the manifestation of hydrogen peroxide enema-induced proctitis in the middle age population which is not a commonly encountered problem for general practitioners. In its early stages, the sigmoidoscopic findings can scarcely be mistaken for another pathologic process such as ulcerative colitis. A history of self-administered enema is crucial. Due to its significant morbidity, physicians should be familiar with the diagnosis and proper management of these patients.

## Case presentation

A 32-year-old female presented with perianal pain and urgency for a few days. The patient stated that she had a history of intermittent constipation. A few days before coming to the hospital, she was constipated and felt some lower abdominal discomfort. She attempted to use an enema which she made from water and hydrogen peroxide. She placed the enema in a vaginal douching bottle. A day following administration, she felt a burning sensation in her rectum with urgency. The concentration of hydrogen peroxide used was unknown. She then developed severe left lower sided cramping abdominal pain followed by passing multiple episodes of watery and foul-smelling stools later in the evening. She also noticed a small amount of bright red blood per rectum after wiping, with mucous discharge and extreme rectal discomfort. 

She had no significant past surgical history besides hemorrhoidectomy. She denied any previous similar episodes, nausea, vomiting, chronic nonsteroidal anti-inflammatory drugs use, any recent changes to her medications, sick contacts or recent international travel. Family history was negative for inflammatory bowel disease or gastrointestinal tract malignancies. No prior endoscopic interventions, such as upper endoscopy or colonoscopy were done.

**Figure 1:**
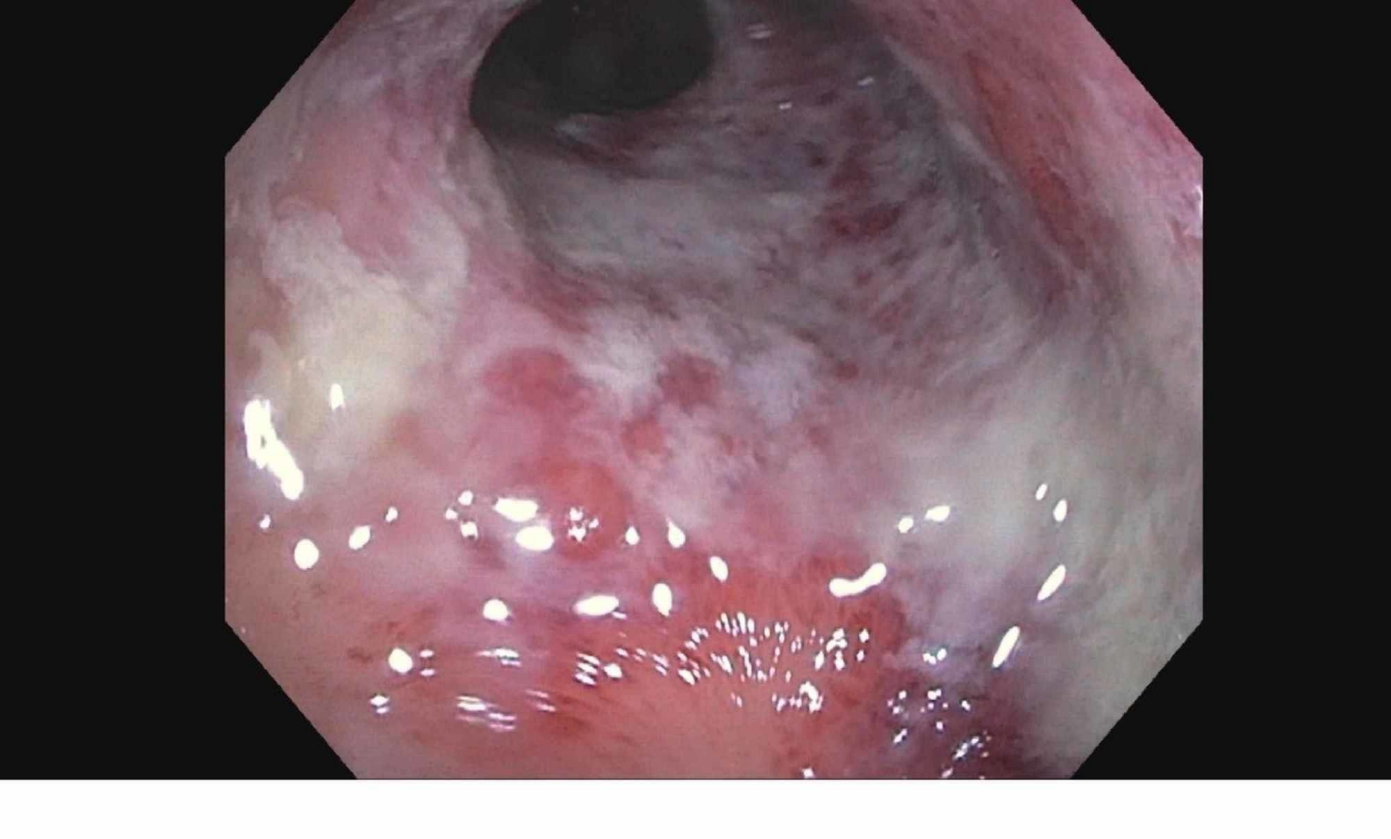
Endoscopic findings showing mucosal friability

**Figure 2:**
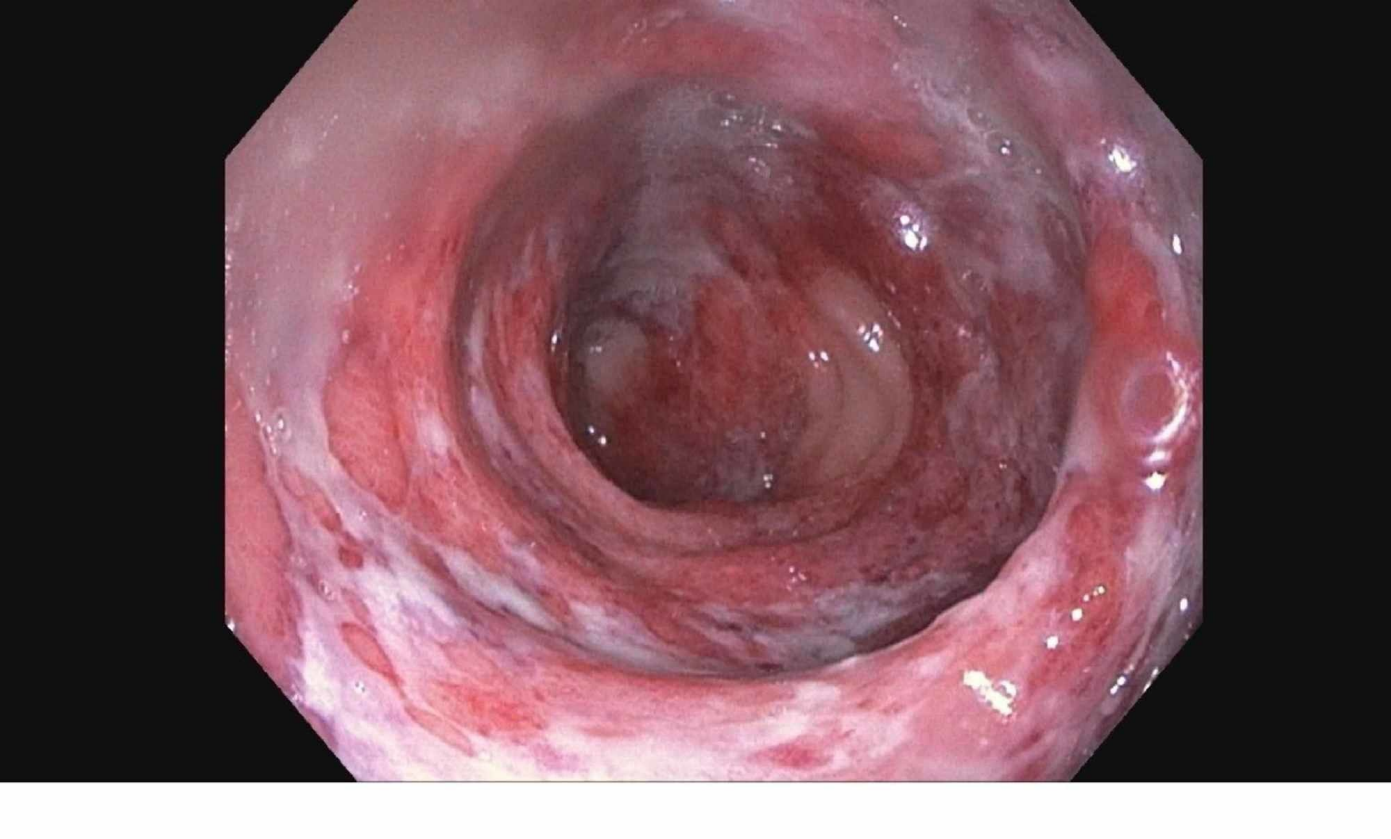
Mucosal friability, purulent exudates, and focal ulcerations after hydrogen peroxide administration

Upon admission, vital signs were within normal limits. She had a soft abdomen on examination, which was mildly tender to deep palpation in the left lower quadrant, and non-distended; no guarding was noted. Bowel sounds were 2+. Labs showed significant neutrophilic leukocytosis with white blood cell count of 16400 with 88% neutrophils, marked elevated C-reactive protein (CRP) 94.5 μg/mL, erythrocyte sedimentation rate (ESR) 41 mm/hr, and fecal calprotectin 634 μg/mg (normal values: 10 to 50 or 60 μg/mg), and negative Clostridium difficile studies. All other stool studies were negative. A complete metabolic profile was done and it was within normal limits. Computed tomography (CT) of the abdomen was done which showed signs of proctitis with inflammatory thickening and edema of the rectosigmoid and rectal vault. Perirectal fluid and fat stranding were also noted. After informed consent was obtained, colonoscopy was done which revealed severe proctitis up to 15 cm from the anal verge manifested by superficial mucosal ulceration, marked erythema, and edema with friable and hemorrhagic mucosa (Figures 1-2).

Rectosigmoid biopsy showed colonic mucosal erosion, acute inflammatory exudate consistent with ulceration, and ischemic type changes. After discussing with the patient, she was given mesalamine which resolved her symptoms. She showed clinical improvement and was discharged home within four days with a complete resolution of her symptoms. She was given mesalamine suppositories nightly at bedtime for two weeks and counseled regarding the discontinuation of enema usage with follow-up in a gastroenterology clinic within two weeks.

## Discussion

Hydrogen peroxide is a disinfectant that has achieved broad applicability in different clinical settings such as an irrigating and disinfecting agent. At one point, it was also popular as a home treatment for constipation given the fact that it is readily available. The pathogenesis of hydrogen peroxide colitis is due to the reaction of producing highly reactive oxygen species causing damage to the colon and rectum mucosa. The chemical damage caused by reactive oxygen species results in reduced blood flow to the large intestine causing rapid distension [4]. This is due to the minute gas cysts formation inside the mucosa and submucosa immediately after exposure. It is then followed by vascular congestion and ulceration. Gas embolism has also been reported because of the gas cysts' penetration into the large veins and lymphatic system. Gas formation in the portal and mesenteric veins results in systemic embolization and peroxidation of lipids [5]. Inflammation of intestine due to hydrogen peroxide can lead to the whitening of the mucosa, otherwise known as the 'snow white’ sign [6].

Clinical manifestations of hydrogen peroxide-induced enteritis range from mild, self-limited colitis to strictures and perforation, sometimes leading to complications and death. The most common presenting symptoms include diarrhea, lower abdominal pain, tenesmus/urgency or rectal bleeding. In one of the cases reported, the patient presented with rapid onset abdominal pain and diarrhea mimicking acute ulcerative colitis leading to shock and death in only four days [7]. In a few cases, findings of acute abdomen with rebound tenderness and rigidity have also been reported [8-9].

Diagnosis of chemical enteritis can be done with CT scan, endoscopy or biopsy. CT scan of the abdomen shows rectal wall thickening, inflammation, and hyperemia consistent with proctitis [10]. Endoscopy findings included wall thickening, mucosal friability, purulent exudates, diffuse and focal ulceration, gray, yellow/green pseudo-membrane formation, necrosis, and perforation of the distal colon or rectum [11]. On biopsy edema, congestion, hemorrhage of lamina propria, destruction of mucosal glands, “bubbly” appearance of goblet cells, ischemic changes, and micro-abscesses with polymorphonuclear leukocytes are seen [12-13].

Management included conservative treatment with bowel rest, fluid resuscitation, and broad-spectrum antibiotics with anaerobic coverage. In some cases, oral and rectal administration of 5-aminosalicylic acid (5-ASA) and corticosteroids can be given [11]. In the literature, some physicians have used parenteral adrenocorticotropic hormone (ACTH) followed by oral prednisone with a good clinical response; most patients recovered after conservative medical therapy [14]. Some serious consequences have been reported including death due to idiopathic hemolytic reaction following plasma transfusion to correct blood loss, portal vein embolism, colonic gangrene, rupture, and fulminant colitis [7,15].

In our case, the diagnosis was made primarily based on patient history and abdominal CT scan, because of the temporal relationship of presenting symptoms following the enema. A confirmatory colonoscopy and biopsy were then done to confirm the diagnosis and assess the extent of the disease.

## Conclusions

In conclusion, patients with unexplained proctitis must be extensively asked about risk factors including the rectal administration of hydrogen peroxide. Clinicians and caregivers should be aware of this chemical culprit. We cannot emphasize enough that hydrogen peroxide must be utilized for external use only and public education on the dangers of hydrogen peroxide enema may be needed.

## References

[1] Olim CB, Ciuti A (1954). Meconium ileus: a new method of relieving obstruction: report of two cases with successful management. Ann Surg.

[2] Pumphrey RE (1951). Hydrogen peroxide proctitis. Am J Surg.

[3] Lee SG, Ko YG, Whang YW, Kim WH (1997). A case of hydrogen peroxidese induced proctitis. J Korean Soc Coloproctol.

[4] Love BL, Siddiqui S, McCallum BJ, Helman RM (2012). Severe chemical colitis due to hydrogen peroxide enema. J Clin Gastroenterol.

[5] Shaw A, Cooperman A, Fusco J (1967). Gas embolism produced by hydrogen peroxide. N Eng J Med.

[6] Bilotta JJ, Waye JD (1989). Hydrogen peroxide enteritis: the “snow white” sign. Gastrointest Endosc.

[7] Sheehan JF, Brynjolfsson G (1960). Ulcerative colitis following hydrogen peroxide enema: case report and experimental production with transient emphysema of colonic wall and gas embolism. Lab Invest.

[8] Ludington LG, Hartman SW, Keplinger JE, Williams FS (1958). Incomplete rupture of the colon following hydrogen peroxide enema. AMA Arch Surg.

[9] Almalouf P, Shehab TM, Daniel AMR, Robinson EA, Barnett JL (2008). Therapeutic hydrogen peroxide enema causing severe acute colitis. Int J Colorectal Dis.

[10] Nunley B, Truss C (2019). On the hot seat: a rare cause of rectal pain. Gastroenterology.

[11] Sheibani S, Gerson LB (2008). Chemical colitis. J Clin Gastroenterol.

[12] Taş A, Aydın YY, Arhan M, Koklu S (2011). Hydrogen peroxide exposure mimicking ulcerative proctitis. Dig Liver Dis.

[13] Jonas G, Mahoney A, Murray J, Gertler S (1988). Chemical colitis due to endoscope cleaning solutions: a mimic of pseudomembranous colitis. Gastroenterology.

[14] Meyer CT, Brand M, DeLuca VA, Spiro HM (1981). Hydrogen peroxide colitis: a report of three patients. J Clin Gastroenterol.

[15] Volonte F, Gervaz P, Poletti PA, Morel P (2010). Portal vein gas embolism following oxygen peroxide enema. Colorectal Dis.

